# The Effect of Cross-Sex Fecal Microbiota Transplantation on Metabolism and Hormonal Status in Adult Rats

**DOI:** 10.3390/ijms25010601

**Published:** 2024-01-02

**Authors:** Andrej Feješ, Paulína Belvončíková, Dafne Porcel Sanchis, Veronika Borbélyová, Peter Celec, Mária Džunková, Roman Gardlík

**Affiliations:** 1 Institute of Molecular Biomedicine, Faculty of Medicine, Comenius University, Sasinkova 4, 811 08 Bratislava, Slovakia; andrej.fejes@fmed.uniba.sk (A.F.); belvoncikova2@uniba.sk (P.B.); veronika.borbelyova@fmed.uniba.sk (V.B.); peter.celec@fmed.uniba.sk (P.C.); 2 Institute for Integrative Systems Biology, University of Valencia and Consejo Superior de Investigaciones Científicas (CSIC), 469 80 Valencia, Spain; dafne.porcel@uv.es (D.P.S.);

**Keywords:** sex differences, testosterone, gut microbiota, microbiome, fecal microbiota transplantation, metabolic disease, cross-sex

## Abstract

Increasing evidence of sexual dimorphism in the pathophysiology of metabolic complications caused by sex steroids is under investigation. The gut microbiota represents a complex microbial ecosystem involved in energy metabolism, immune response, nutrition acquisition, and the health of host organisms. Gender-specific differences in composition are present between females and males. The purpose of this study was to use cross-sex fecal microbiota transplantation (FMT) for the detection of sex-dependent metabolic, hormonal, and gut microbiota changes in female and male recipients. Healthy non-obese female and male Wistar rats were divided into donor, same-sex, and cross-sex recipient groups. After a 30-day period of FMT administration, biochemical markers (glucose and lipid metabolism) and sex hormones were measured, and the gut microbiota was analyzed. The cross-sex male recipients displayed a significantly lower testosterone concentration compared to the males that received same-sex FMT. Sex-dependent changes caused by cross-sex FMT were detected, while several bacterial taxa correlated with plasma testosterone levels. This study represents the first to study the effect of cross-sex changes in the gut microbiome concerning metabolic and hormonal changes/status in adult non-obese Wistar rats. Herein, we present cross-sex FMT as a potential tool to modify sex-specific pathologies.

## 1. Introduction

The prevalence of metabolic diseases, such as obesity, metabolic syndrome, and type 2 diabetes, is increasing worldwide [1,2,3]. There is evidence of sexual dimorphism in the metabolic complications caused by sex hormones, such as estrogens and androgens [4,5,6]. The gut microbiota plays a crucial physiological role in the local and systemic immune response and energy extraction. Dysbiosis of the gut microbiota appears to be involved in various diseases, including metabolic disorders [7]. The overall incidence of obesity is higher in women compared to men, with increasing age resulting in abdominal fat gain. Moreover, the prevalence of metabolic syndrome is also greater in women, while the incidence of type 2 diabetes (T2D) is reversed. In men, a higher prevalence of T2D occurs before puberty, while in women it occurs after menopause [8,9,10]. These differences in the development of obesity are supported by differences in fat distribution. While in men and postmenopausal women, fat is distributed in an abdominal (visceral) fat pattern, in premenopausal women, it is a typical peripheral (subcutaneous) distribution pattern. Both patterns are strongly regulated and related to sex steroid changes during life in both genders [11]. The physiological changes in sex steroid concentrations after menopause lead to fat redistribution [12], while androgen deprivation in men results in increased adiposity in a visceral fat mass [13]. Testosterone supplementation in non-obese men with androgen deficiency leads to a decrease in visceral adiposity, improved glycemic control, and insulin sensitivity [14,15]. The gut microbiota represents a complex microbial ecosystem strongly related to nutrition acquisition, energy metabolism, immune response, and regulating the metabolic health of the host [16,17,18]. The role of gut microbiota in metabolic disorders has been investigated in recent years [19,20,21]. Gender-specific differences in gut microbiota composition are evident between males and females [22]; however, the effect of cross-sex fecal microbiota transplantation (FMT) on these differences has not yet been elucidated. Research on this specific topic is limited, and little is known about the susceptibility of both sexes to receive a transplant or the ability of the sex-specific transplant to colonize and rebuild the donor ecosystem. The present study hypothesizes that the cross-sex replacement of the gut microbiome might change the hormonal and metabolic status in non-obese, healthy female and male rats. Therefore, obesity and metabolic markers (body weight, glycemia, cholesterol, AST, uric acid), sex hormones, and the gut microbiome composition of female and male rats that served as donors of FMT were assessed in a cross-sex and same-sex manner and compared with the hormonal and metabolic status of FMT recipient rats. The majority of experimental studies use FMT as a therapeutic tool to restore the gut microbiota in animals with a disease (FMT from healthy to diseased) or as a tool to induce the pathological phenotype in healthy individuals (FMT from diseased to healthy). However, little is known about “healthy to healthy” FMT in terms of its effect on metabolism and/or hormone levels. To the best of our knowledge, this paper is the first to study the effect of cross-sex changes in the gut microbiome concerning metabolic and hormonal changes/status in adult non-obese Wistar rats.

## 2. Results

### 2.1. Body Weight and Food Intake

**Figure 1:**
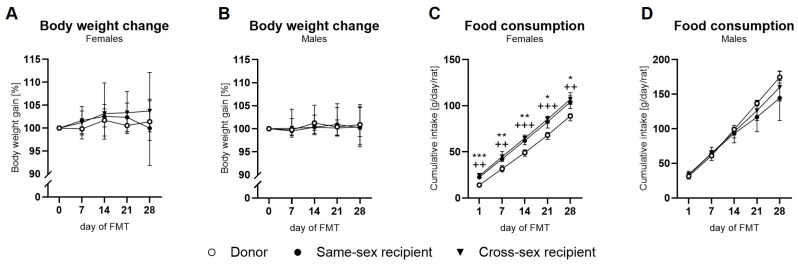
Body weight change in female (**A**), and male (**B**) donor and recipient animals and food consumption in female (**C**) and male (**D**) donor and recipient animals. Data are presented as mean ± SEM. *—differences between same-sex recipient female rats and donors; +—differences between cross-sex female rats and donors; * *p* < 0.05; ** *p* < 0.01; *** *p* < 0.001; ++ *p* < 0.01; +++ *p* < 0.001.

The body weight gain in female rats increased over time (time: F(4,64) = 3.839; *p* < 0.05) in all the experimental groups, while the effect of FMT was not observed (F(2,16) = 0.67; *p* = ns) (Figure 1A). In males, the body weight did not change during the FMT administration (time: F(4,68) = 1.34; *p* = ns; FMT: F(2,17) = 0.088; *p* = ns, Figure 1B). The cumulative food intake in the female rats was increased during the FMT administration in all the experimental groups (time: F(4,36) = 1476; *p* < 0.001; FMT: (F(2,9) = 38.37; *p* < 0.001). Both the same-sex recipient and cross-sex recipient female rats consumed significantly more food during the FMT administration (day 1, day 7, day 14, day 21, day 28; all: *p* < 0.05 for same-sex and cross-sex recipient females) in comparison to the donor female rats (Figure 1C). The donor female rats consumed less food during the FMT administration compared to both the same-sex recipient (day 1, day 7, day 14, day 21, day 28; all: *p* < 0.05) and cross-sex recipient female rats (day 1, day 7, day 14, day 21, day 28; all: *p* < 0.05, Figure 1C). The cumulative food consumption in the male rats increased over time (F(4,32) = 368.1; *p* < 0.001), but a significant effect of the FMT on the food consumption was not detected (F(2,8) = 1.067; *p* = ns, Figure 1D). Regarding the terminal body weight, a significant effect of sex (F(1,33) = 198; *p* < 0.001) was observed: the donor females displayed a lower body weight compared to the donor males (t(33) = 6.765; *p* < 0.001), the same-sex recipient females displayed a lower body weight than the same-sex recipient males (t(33) = 8.747; *p* < 0.001), and the cross-sex recipient females had a lower body weight than the cross-sex recipient males (t(33) = 9.084; *p* < 0.001, Figure 1A). FMT did not affect the terminal body weight of the rats (F(2,33) = 0.373; *p* = ns, Figure 1A).

### 2.2. Metabolic Parameters

**Figure 2:**
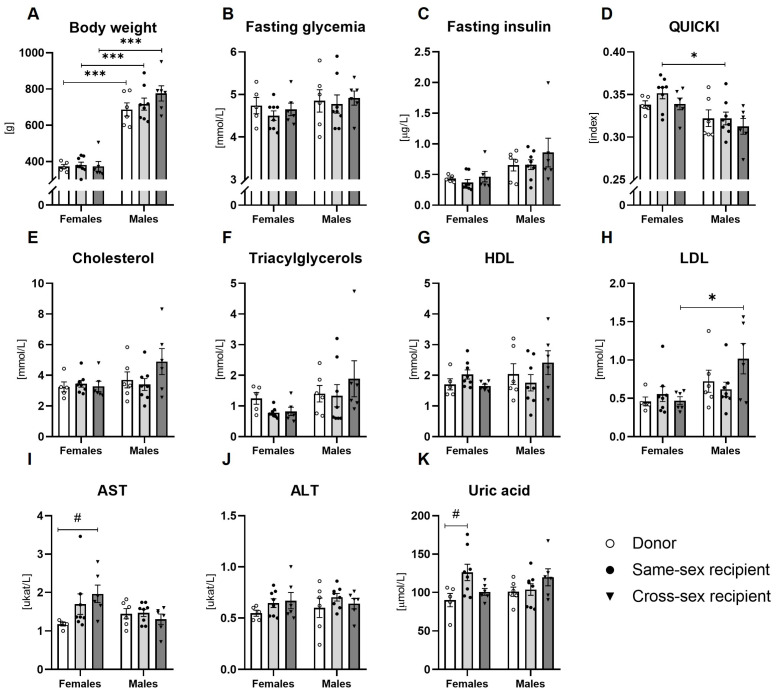
Biochemical analysis of plasma: (**A**) Body weight; (**B**) Fasting glycemia; (**C**) Fasting insulin concentrations; (**D**) Quantitative index of insulin sensitivity (QUICKI); (**E**) Total cholesterol concentration; (**F**) Concentration of triacylglycerols; (**G**) HDL cholesterol concentrations; (**H**) LDL cholesterol concentrations; (**I**) AST concentration; (**J**) ALT concentration; (**K**) Uric acid concentration. Data are presented as mean ± SEM. *—sex difference; #—the difference between donor, same-sex, and cross-sex rats in individual sex groups; * *p* < 0.05; *** *p* < 0.001; # *p* < 0.05; HDL—high-density lipoproteins; LDL—low-density lipoproteins; AST—aspartate aminotransferase; ALT—alanine aminotransferase.

Two-way ANOVA showed the main effect of sex on the fasting insulin concentrations (F(1,33) = 10.64; *p* < 0.01), a quantitative index of insulin sensitivity (QUICKI; F(1,33) = 14.02; *p* < 0.001), circulating LDL (F(1,33) = 8.94; *p* < 0.01), and triacylglycerol concentrations (F(1,33) = 5.142; *p* < 0.05). Fasting glycemia, plasma total cholesterol and HDL cholesterol concentrations, AST, ALT, and uric acid concentration were not affected by sex (all: *p* = ns). FMT did not affect any of the metabolic parameters (all: *p* = ns, Figure 2B–K).

### 2.3. Testosterone

**Figure 3:**
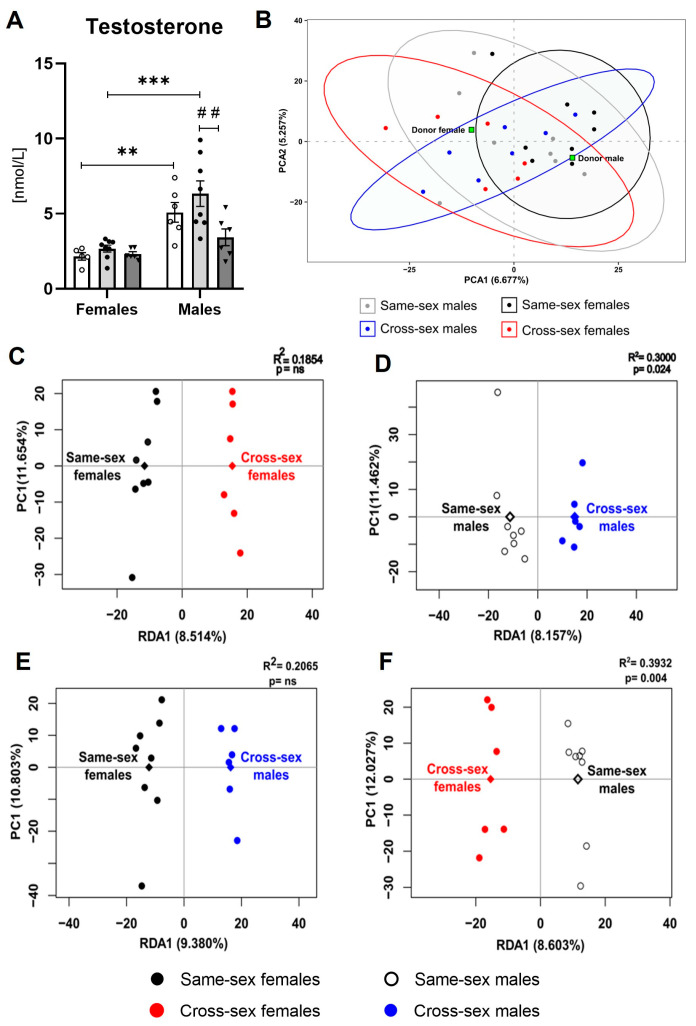
Plasma testosterone concentrations and gut microbiota composition analysis: (**A**) Plasma testosterone concentrations; (**B**) Principal component analysis of the gut microbiota—all recipients; (**C**) Redundancy analysis of all female recipients according to different FMT donor sex (same-sex females, cross-sex females); (**D**) Redundancy analysis of all male recipients according to different FMT donor sex (same-sex males, cross-sex males); (**E**) Redundancy analysis of all mice receiving FMT from female donors (same-sex females, cross-sex males); (**F**) Redundancy analysis of all mice receiving FMT from male donors (same-sex males, cross-sex females). Data are presented as mean ± SEM. *—sex difference; #—the difference between donor, same-sex, and cross-sex rats in individual sex groups; ** *p* < 0.01; *** *p* < 0.001; ## *p* < 0.01.

The plasma testosterone concentrations showed sex differences, with higher testosterone concentrations in the males (F(1,33) = 30.13; *p* < 0.001) than in the females. The female donors displayed lower concentrations of testosterone compared to the male donors (t(33) = 3.361; *p* < 0.01). Two-way ANOVA indicated a significant effect of 30-day-long FMT application on the circulating testosterone concentration (F(2,33) = 4.455; *p* < 0.05). The same-sex recipient females displayed lower concentrations of plasma testosterone compared to the same-sex recipient males (t(33) = 5.089; *p* < 0.001), while no sex differences between the cross-sex females and males were detected (t(33) = 1.335; *p* = ns). Lower testosterone concentrations were detected in the cross-sex male recipients compared to the same-sex male recipients (t(33) = 3.738; *p* < 0.01, Figure 3A).

### 2.4. Corticosterone

The plasma corticosterone concentrations showed sex differences, with higher corticosterone concentrations in the females (F(1,33) = 22.10; *p* < 0.001) than in the males. No effect of transplantation was shown as no differences were detected between the same-sex and cross-sex recipients in both the male and female groups (Supplementary Figure S1).

### 2.5. Gut Microbiome

We analyzed the 16S rRNA gene amplicon sequencing data of the gut microbiome samples in the female and male recipients that received either same-sex or cross-sex FMT as well as the donor samples. Alpha diversity analysis, represented by the Shannon index, indicated no variations in microbial richness and evenness of the same-sex females, cross-sex females, same-sex males, and cross-sex females (*p* = ns). Similarly, the initial principal component analysis (PCA) did not show any specific clustering patterns for the samples corresponding to the four recipient groups when the microbial composition was based on the ASV level (Figure 3B), neither on the phylum, class, order, family, or genus levels. Importantly, the female and male donor pool samples were clearly separated from each other, suggesting sex-based microbiome composition differences in the donor mice. To examine any minor compositional variations between the groups, we performed a redundancy analysis (RDA). The redundancy analysis revealed gut microbiota differences in various bacterial groups across the donors and four recipient groups: same-sex males, cross-sex males, same-sex females, and cross-sex males (day 30). In all four recipient groups, bacteria belonging to class Bacteroidia (*p* < 0.05), the orders Bacteroidales, Gastranaerophilales, and Clostridiales vadin BB60 group (*p* < 0.05), the classes Lachnospiraceae, Muribaculaceae, and Oscillospiraceae (*p* < 0.05), and the genera *Helicobacter*, Lachnospiraceae UCG-008 group, Lachnospiraceae NK4A136 group, Prevotellaceae Ga6A1 group, and Prevotellaceae NK3B31 group (*p* < 0.05) showed diverse distribution according to the RDA.

The RDA results revealed specific group clustering of individual samples and the segregation of the four recipient groups, suggesting a potential association between specific microbial communities and sex (R^2^ = 0.2921, *p* < 0.05, permutation test; Supplementary Figure S2). To obtain a deeper insight into the sex-based differences in FMT, we performed additional RDA for sub-groups: mice receiving FMT from female donors (same-sex recipient females and cross-sex recipient males), mice receiving FMT from male donors (same-sex recipient males and cross-sex recipient females), all female recipients (receiving FMT from females—same-sex females, and receiving FMT from males—cross-sex females), and all male recipients (receiving FMT from males—same-sex males, and receiving FMT from females—cross-sex males). The results suggest that the FMT was successfully transplanted in the case of the male donors, showing that the same-sex males and cross-sex females that received male FMT had significantly different microbiome compositions (R^2^ = 0.3932, *p* < 0.01), where the ASVs belonging to the orders Bacilli RF39, Clostridia UCG-014, Clostridia vadin BB60, and Gastranaerophilales, the families Lachnospiraceae and Oscillospiraceae, and the genera *Alistipes*, *Oscillibacter*, and Oscillibacteraceae NK4A214 group (*p* < 0.01) were enriched in the female recipients, and the ASVs belonging to the class Bacteroidia, order Bacteroidales (*p* ≤ 0.001), family Lactobacillaceae, and genus *Lactobacillus* (*p* ≤ 0.01) were enriched in the male recipients (Figure 3D). In contrast, the same-sex females and cross-sex males who received female FMT did not differ significantly by their microbiome composition (R^2^ = 0.2065, *p* = ns; Figure 3C). Accordingly, the male recipients reacted differently to FMT from male and female donors (R^2^ = 0.3000, *p* < 0.05). The microbiome of the male recipients that received FMT from males was characterized by an increased proportion of ASVs belonging to the class Bacteroidia, family Lachnospiraceae and unidentified ASV_285 bacteria (*p* ≤ 0.001), order Bacteroidales, and genera Lachnospiraceae NK4A136 group and *Eubacterium coprostanoligenes* group (*p* < 0.01). The male recipients that received FMT from females contained an increased proportion of unidentified ASV_314 bacteria (*p* < 0.001) and ASVs belonging to the orders Bacilli RF39, Clostridia UCG-014, Bacteroidales, and Gastranaerophilales and the genera *Streptococcus, Frisingicoccus*, and Family XIII AD3011 group (*p* < 0.01; Figure 3F). In contrast, the female recipients maintained nearly the same microbiome composition, independent of whether they received FMT from females or males (R^2^ = 0.1854, *p* = ns; Figure 3E).

**Figure 4:**
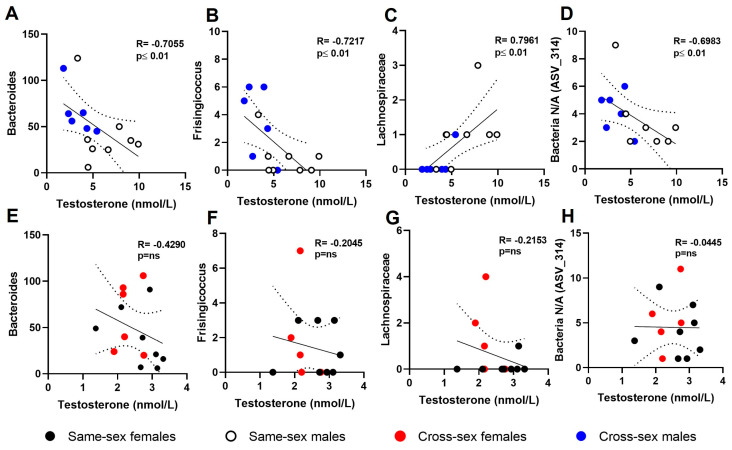
Correlation of testosterone concentration with specific bacterial species: (**A**) Correlation between plasma testosterone concentration and Bacteroides abundance in recipient males; (**B**) Correlation between plasma testosterone concentration and *Frisingicoccus* abundance in recipient males; (**C**) Correlation between plasma testosterone concentration and Lachnospiraceae abundance in recipient males; (**D**) Correlation between plasma testosterone concentrations and Bacteria N/A (ASV_314) in recipient males; (**E**) Correlation between plasma testosterone concentration and Bacteroides abundance in recipient females; (**F**) Correlation between plasma testosterone concentration and *Frisingicoccus* abundance in recipient females; (**G**) Correlation between plasma testosterone concentration and Lachnospiraceae abundance in recipient females; (**H**) Correlation between plasma testosterone concentrations and Bacteria N/A (ASV_314) in recipient females.

To reveal the specific ASVs that could be associated with plasma concentrations of testosterone (TST), Spearman correlation analysis was performed. Several bacterial species showed positive or negative correlations with plasma concentrations of TST. In the males, the abundance of Bacteroides negatively correlated with the TST levels (including same-sex and cross-sex males, R = −0.7055; *p* < 0.01; Figure 4A), while in the females, this effect was not observed (same-sex females and cross-sex females, R = −0.429; *p* = ns; Figure 4B). The circulating concentrations of TST in the same-sex recipient females and cross-sex recipient males negatively correlated with the proportion of *Frisingicoccus* (R = −0.7217; *p* < 0.01; Figure 4C), while in the female recipient groups, we did not observe any significant association between TST and *Frisingicoccus* abundance (R = −0.2045; *p* = ns; Figure 4D). The proportion of Lachnospiraceae positively correlated with the plasma TST concentration in the male recipient groups (R = 0.7961; *p* < 0.01; Figure 4E), while in the female recipients, no significant association was observed (R = −0.2153; *p* = ns; Figure 4F). The proportion of a specific unidentified ASV_314 showed a negative correlation with the plasma TST concentrations in the male recipients (R = 0.6983; *p* < 0.01; Figure 4G) but not in the female recipients (R = −0.0445; *p* = ns; Figure 4H).

## 3. Discussion

Sexual dimorphism plays a crucial role in the pathogenesis of various metabolic disorders; therefore, the pathophysiological role of sex steroids has been studied in recent years. Sex differences are evident in the prevalence and severity of metabolic disorders, including obesity and metabolic syndrome [23,24,25,26]. The role of biological sex in the pathophysiology of metabolic disorders is still not fully understood. Interestingly, the gut microbiota plays an important role in host metabolism and immunity [27]. Gut microbiota dysbiosis is associated with the development of numerous disorders. Recently, treatment based on modification of the gut microbiota has been proposed and investigated in many immune, auto-immune, and metabolic diseases [28,29,30,31]. Fecal microbiota transplantation (FMT) seems to be one of the tools used to therapeutically shape gut microbiota. Various components of metabolism, including energy balance and the metabolism of glucose and lipids, are controlled in a sexually dimorphic manner. In the present study, we have shown that sex differences in non-obese healthy rats are in body weight, insulin sensitivity, and plasma lipid markers (LDL, triacylglycerols). Sex differences in glucose and lipid metabolism have been previously researched [4,32]. FMT in same-sex and cross-sex recipient females and males did not affect the sex differences in the above-mentioned metabolic components. However, the plasma testosterone concentrations differed between the female and male donor and same-sex recipient subjects. Interestingly, there was no sex difference between the cross-sex recipient female and male animals. The male rats that received FMT from female donors displayed lower plasma concentrations of testosterone compared to the male recipients that received same-sex FMT. This happened likely without affecting the hypothalamic–pituitary–adrenal axis, as no differences in the corticosterone concentrations between the same-sex and cross-sex recipients of both sexes were shown. Sex hormones, such as estrogens, progesterone, and testosterone, are major determinants of the differences between sexes in mammals. Females and males displayed same-sex hormone types but with different organ production, concentrations in the blood, and interactions with the target organ [33]. Ongoing discussion is focused on the role of the interaction of sex hormones with the composition of the gut microbiota and its function in sexual differences in many disorders. These findings suggest the important role of sex steroids in the composition of the gut microbiota. It was shown that colon tissue can express molecules involved in steroidogenesis and, subsequently, the synthesis and metabolism of testosterone [34]. We showed that a higher abundance of *Bacteroides* in the male rats that received same-sex and cross-sex FMT was associated with lower plasma testosterone concentrations. It was previously shown that higher Porphyromonadaceae (phylum Bacteroidetes) abundance in the gut microbiota composition was detected in control females and males following castration. Moreover, castrated adult male mice showed the same gut microbiota composition compared to the adult female mice [35]. On the other hand, in both the recipient female groups, we did not observe any association between *Bacteroides* abundance and testosterone concentration. In the present study, a higher abundance of the Lacchnospiraceae (phylum Firmicutes) in the males was associated with higher plasma concentrations of testosterone. The Firmicutes can promote the synthesis of testosterone and boost the host organism [36]. In ovariectomized females compared to control males, Kaliannan et al. observed a decrease in the abundance of the Proteobacteria phylum, a decrease in the Firmicutes/Bacteroides ratio, a higher *Bifidobacterium/Enterobacteriacea* ratio, and an increase in the abundance of *Akkermansia* (phylum Verrucomicrobia) [37]. It was previously shown that the concentration of testosterone in patients with T2D was negatively associated with the abundance of Lachnospirales and Firmicutes [38]. We have shown that a higher abundance of the genus *Frisingicoccus* (phylum Firmicutes, class Clostridia, order Lachnospirales, family Lachnospiraceae) was associated with lower plasma testosterone concentrations in the male recipients. In contrast, the Lachnospiraceae at the family level positively correlated with the plasma testosterone concentrations. These results suggest the importance of gut microbiota composition investigation at all species levels concerning sex steroids. Cross-sex FMT in male recipients can promote a decrease in plasma testosterone concentrations. The commensal microbiota community can potentially affect sex hormones produced by enzymatic activity in the intestine. It was shown that the gut microbiota is involved in the deglucuronidation of dihydrotestosterone and testosterone, resulting in high concentrations of androgens in the host organism [39]. In the current study, a specific unidentified ASV_314 was detected, which was also identified by another research team [40]. This unidentified bacterial ASV_314 abundance negatively correlated with plasma testosterone concentrations in males. All the identified bacterial species (*Bacteroides*, Lacchnospiraceae, *Frisingicoccus*) were associated with plasma testosterone concentrations just in the male same-sex and cross-sex recipients, with clear clustering of animals according to FMT. The clustering and association of the bacterial species involved in the testosterone changes were not significant in the female recipients (same-sex or cross-sex FMT recipients). These findings point out the sex difference in the ability and effectiveness of FMT between female and male recipient rats. The decline in the abundance of *Bacteroides*, *Prevotella*, *Desulfovibrio*, *Lactobacillus*, and *Oxalobacter* genera diversity in the gut is related to the higher prevalence of chronic metabolic disorders. In both lean and obese individuals, gut dysbiosis and low richness in gut microbial abundance are associated with higher adiposity, insulin resistance, dyslipidemia, and low-grade inflammation [41]. Gender differences in gut microbiome diversity have been reported, with a higher abundance of *Bacteroides* and *Prevotella* genera in males compared to females [42]. Androgens regulate the sex-specific gut dysbiosis in obese female and male mice, resulting in a decline in glucose metabolism driven by *Prevotella* sp. *Bacteroides massiliensis* and *Cupriavidus metallidurans* [21]. In addition, androgen-deficient obese mice showed sex-dependent development of metabolic dysfunction linked to enriched gut diversity with *Turicibacter* and *Lactobacillus reuteri* that caused early death in male mice [43]. Moreover, estrogen treatment in high-fat-diet-fed mice showed a modest impact on the gut microbiome diversity, resulting in an increased abundance of *Collinsella aerofaciens* F in males [44]. Thus, the gut microbiota composition might be partially responsible for sex differences in sex hormones and the prevalence and severity of metabolic diseases.

## 4. Materials and Methods

### 4.1. Animals

Female (n = 19) and male (n = 21) Wistar rats at the age of 10 months were purchased from Velaz (Charles River, Velaz, Prague, Czech Republic). All the animals were maintained under the standard conditions (temperature 25 ± 2 °C and humidity 55 ± 10%) with a 12:12 h light–dark cycle and ad libitum access to standard chow (KMK20, EYPY, Czech Republic) and water. The rats were group-housed (2–3 rats per cage) in polycarbonate cages (open caging system, 50 × 36 × 19 cm) with bedding (Safe Select Fine, Velaz, Prague, Czech Republic) and polycarbonate enrichment for rats—tunnels (Velaz, Prague, Czech Republic). All the experimental procedures were approved by the Ethical Committee of the Institute of Molecular Biomedicine, Comenius University, Bratislava and were conducted following EU Directive 2010/63/EU and Slovak legislation.

### 4.2. Experimental Groups

**Figure 5:**
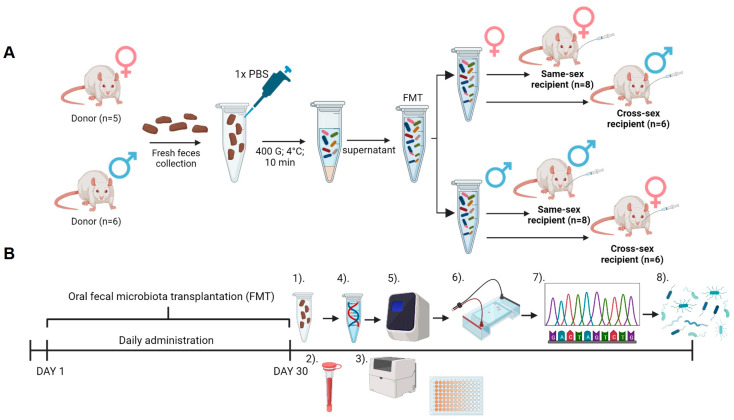
Experimental design: (**A**) Preparation of fecal microbiota transplantation (FMT) and experimental groups. (**B**) Timeline of the experiment—(1)—Recipient sample collection; (2)—Blood collection from the tail vein and plasma preparation; (3)—Biochemical analysis of plasma samples; (4)—Isolation of bacterial DNA from stool samples; (5)—DNA analysis and quantity control and amplification of 16S rRNA V4 regions; (6)—Endpoint Ruby Taq electrophoresis; (7)—Sample sequencing using MiSeq instrument; (8)—Data processing. Created with BioRender.com.

The animals were divided into experimental groups according to a received vehicle: PBS (female donor: n = 5; male donor: n = 6), FMT from the same sex (female same-sex recipient: n = 8; male same-sex recipient: n = 8), and FMT from the opposite sex (females cross-sex recipient: n = 6; males cross-sex recipient: n = 7). Animals from the same experimental group were housed together. The body weight and food consumption were measured weekly (Figure 5A).

### 4.3. Fecal Microbiota Transplantation and Recipient Sample Collection

For the preparation of the FMT solution, freshly collected feces were used. Fresh fecal boluses were placed into 15 mL falcon tubes under sterile conditions from every donor animal separately. The feces samples were weighed and 1.5 g from every bolus was pooled. The pooled feces were dissolved in 1X PBS and immediately centrifuged (400× *g*, 4 °C, 10 min). The supernatant was collected and used as FMT. For the administration of FMT, oral gavage was used in a volume of 100 µL per animal [45,46]. The animals received FMT daily for 30 days (Figure 5A). Feces samples from the recipients were collected individually at the end of the experiment under sterile conditions and immediately frozen at −80 °C (Figure 5B).

### 4.4. Blood Analysis

Following a 30-day-long FMT, blood collection was performed. Before blood collection, the animals were fasted overnight. The fasting glycemia was measured using a glucometer (Accu Chek Performa, Roche Slovakia, s.r.o Diabetes Care, Bratislava, Slovakia). Blood was collected from the tail vein into the EDTA collection tube (K3 EDTA, Microvette, Sarstedt, Nümbrecht, Germany). The blood was centrifuged for 10 min at 1600× *g* and 4 °C, and the plasma supernatant was stored at −20 °C until further analysis. Besides measuring the concentration of fasting insulin (Rat Insulin ELISA kit, Mercodia, Uppsala, Sweden), the plasma samples were used to measure the circulating concentrations of triglycerides (TAG), total cholesterol (CHOL), high-density lipoproteins (HDL), low-density lipoproteins (LDL), aspartate aminotransferase (AST), and alanine aminotransferase (ALT) using a Biolis 24i Premium analyzer (Tokyo Boeki Machinery, Tokyo, Japan). The plasma testosterone was measured using the ELISA method (DRG Diagnostic, Marburg, Germany) (Figure 5B). The plasma corticosterone was measured using an ELISA kit (DRG Diagnostic, Marburg, Germany). From the fasting glycemia and fasting insulin concentrations, the quantitative index of insulin sensitivity (QUICKI) was calculated [47].

### 4.5. Microbiome Analysis

Bacterial DNA was extracted using a commercial kit (QIAamp Fast DNA Stool Mini kit, Qiagen, Hilden, Germany), following the manufacturer’s instructions. The DNA quality and quantity control were verified by spectrophotometric determination (Qubit dsDNA HS Assay Kit, Thermo Fisher Scientific Inc., Waltham, MA, USA). The variable V4 region of the 16S rRNA gene was amplified using the modified 515F forward primer 5′ GTGYCAGCMGCCGCGGTAA 3′ and the 806R reverse primer 5′ GGACTACNVGGGTWTCTAAT 3′ [48,49], also used in the protocol for paired-end 16S rRNA sequencing on the Illumina platform by the Earth’s Microbiome Project [50]. The PCR mixture included the following: 10 µL of Ruby Taq Master Mix polymerase (Jena Bioscience), 5 µL of extracted bacterial DNA, and 0.5 µL of each primer (200–400 nM). The total reaction volume was 20 µL. The PCR conditions were 95 °C for an initial 5 min, followed by 28 cycles of 95 °C for 30 s, 50 °C for 45 s, and 72 °C for 60 s. The quality and quantity of the amplified DNA were analyzed using 1.5% gel electrophoresis with 2 µL of Gel Loading Dye (New England BioLabs, Inc., Ipswich, MA, USA) and a 100 bp DNA Ladder (New England BioLabs, Inc.) (Figure 5B). The samples were pair-end sequenced on the MiniSeq Sequencing System of the Illumina platform (ID MN01227) at the Research Resources Center (MC937), University of Illinois in Chicago, IL. The sequenced raw reads can be found under project accession PRJEB70472 in ENA.

### 4.6. Data Processing and Results Analysis

The quality of the paired-end Illumina sequences was checked using FastQC v.0.11.9 [51] and MultiQC v.1.13 [52]. The primer sequences were removed by Cutadapt v3.5100 [53] using the linked behavior, an overlap of 10 nucleotides, removing reads of length zero, and discarding untrimmed sequences. Afterwards, the sequence processing was continued using DADA2 v1.22.0101 [54], which is the core of the pipeline. This package is used for trimming sequence ends to remove low-quality ends as well as for ASV generation and chimera removal. The sequence quality profiles were inspected again using FastQC v.0.11.9 [51] and MultiQC v.1.13 [52], and quality trimming was carried out accordingly. The ‘truncLen’ parameter was set at 132 bp for the R1 and R2 files. The filtering out of low-quality R1 and R2 reads was performed using a max expected error of 2 and 5 respectively, with the rest of the default parameters. The paired-end reads were merged to reconstruct the amplicon sequence variants (ASVs) using the DADA2 core sample inference algorithm with the pool inference behavior. Subsequently, the chimeric sequences were removed using the consensus method of the DADA2 package. Finally, the 16S rDNA V4 gene sequences were classified by the IDTAXA classifier of the DECIPHER v2.22108 [55] package with 50% confidence using the SILVA SSU r138 (modified) database implemented in the IDTAXA classifier (Figure 5B). The quality of the paired-end Illumina sequences was checked using FastQC v.0.11.9 [51] and MultiQC v.1.13 [52]. The primer sequences were removed by Cutadapt v3.5100 [53] using the linked behavior, an overlap of 10 nucleotides, removing reads of length zero, and discarding untrimmed sequences. Afterwards, the sequence processing was continued using DADA2 v1.22.0101 [54], which is the core of the pipeline. This package is used for trimming sequence ends to remove low-quality ends as well as for ASV generation and chimera removal. The sequence quality profiles were inspected again using FastQC v.0.11.9 [51] and MultiQC v.1.13 [52], and quality trimming was carried out accordingly. The ‘truncLen’ parameter was set at 132 bp for the R1 and R2 files. The filtering out of low-quality R1 and R2 reads was performed using a max expected error of 2 and 5 respectively, with the rest of the default parameters. The paired-end reads were merged to reconstruct the amplicon sequence variants (ASVs) using the DADA2 core sample inference algorithm with the pool inference behavior. Subsequently, the chimeric sequences were removed using the consensus method of the DADA2 package. Finally, the 16S rDNA V4 gene sequences were classified by the IDTAXA classifier of the DECIPHER v2.22108 [55] package with 50% confidence using the SILVA SSU r138 (modified) database implemented in the IDTAXA classifier (Figure 5B).

### 4.7. Statistical Analysis

For the statistical evaluation and visualization of the body weight gain, food consumption, and biochemical parameters, GraphPad Prism version 8.0.1 (GraphPad Software, Inc., San Diego, CA, USA) was used. Two-way ANOVA was performed with independent factors: sex and fecal microbiota transplantation (FMT) with the Bofenroni post-hoc test. For the statistical evaluation of the microbiome data, RStudio version 4.2.1 served as the primary software tool. Initially, sequences with less than one count were removed, and the data were normalized. The Shannon index, principal component analysis (PCA), and redundancy analysis (RDA) were obtained using vegan and ggplot2 libraries. RDA was performed with a 1000 permutations test. Spearman correlations of the bacterial species with testosterone concentrations were analyzed in JASP software version 0.18.1 and visualized in GraphPad Prism 8.0.1 (GraphPad Software, Inc., San Diego, CA, USA).

## 5. Conclusions

Although several recommendations have been previously proposed for FMT as a therapeutic tool, the sex of the donor has not been investigated as one of the factors determining the effect of FMT on the recipient. Thus, the results from the current study have implications for FMT procedures. It seems that cross-sex FMT does not affect the metabolic components. However, male recipients of a female transplant display lower plasma testosterone levels, which might play a role in designing future FMT-based therapy for sex hormone-mediated pathologies. Moreover, the present study can help to understand the biological processes behind FMT, which might have implications in future clinical studies using FMT as a preventive (instead of therapeutic) strategy. To clarify these phenomena, further experimental studies need to be performed.

## Data Availability

Data are contained within the article and supplementary materials.

## Supplementary Materials

The following supporting information can be downloaded at https://www.mdpi.com/article/10.3390/ijms25010601/s1.
