# Supplementary figures and images for: The Effect of Cross-Sex Fecal Microbiota Transplantation on Metabolism and Hormonal Status in Adult Rats
Source: Int J Mol Sci. 2024 Jan 2;25(1):601. doi: 10.3390/ijms25010601 (PMC10778742; doi:10.3390/ijms25010601)

# Corticosterone

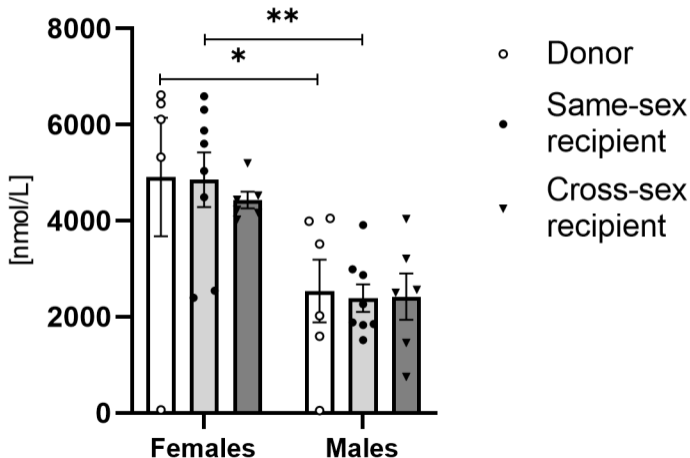

# RDA triplot – all recipients

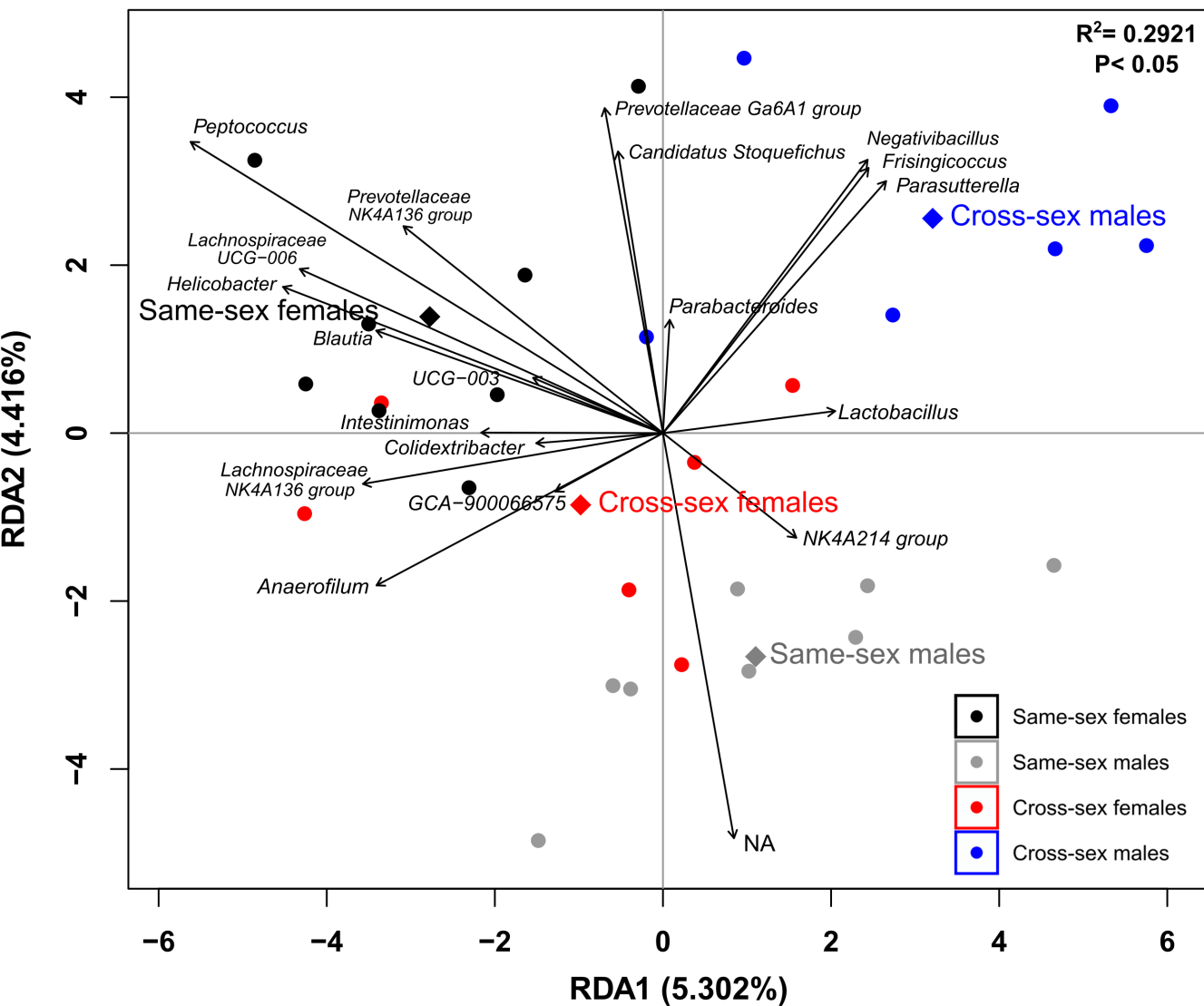

Supplement: Supplementary file 1 [file ijms-25-00601-s001.zip › ijms-2776540-supplementary.pdf]
